# Voltage-independent sodium channels emerge for an expression of activity-induced spontaneous spikes in GABAergic neurons

**DOI:** 10.1186/1756-6606-7-38

**Published:** 2014-05-20

**Authors:** Wei Lu, Bo Wen, Fengyu Zhang, Jin-Hui Wang

**Affiliations:** 1 State Key lab for Brain and Cognitive Sciences, Institute of Biophysics, Chinese Academy of Sciences, 15 Datun Road, Beijing 100101, China; 2 Graduate School of the Chinese Academy of Sciences, Beijing 100049, China; 3 Qingdao University, Medical College, 38, Dengzhou, Shandong 266021, China

**Keywords:** Action potential, Spontaneous spikes, Threshold potential, Sodium channel, Hippocampus, GABAergic neurons

## Abstract

**Background:**

Cerebral overexcitation needs inhibitory neurons be functionally upregulated to rebalance excitation vs. inhibition. For example, the intensive activities of GABAergic neurons induce spontaneous spikes, i.e., activity-induced spontaneous spikes (AISS). The mechanisms underlying AISS onset remain unclear. We investigated the roles of sodium channels in AISS induction and expression at hippocampal GABAergic neurons by electrophysiological approach.

**Results:**

AISS expression includes additional spike capability above evoked spikes, and the full spikes in AISS comprise early phase (spikelets) and late phase, implying the emergence of new spikelet component. Compared with the late phase, the early phase is characterized as voltage-independent onset, less voltage-dependent upstroke and sensitivity to TTX. AISS expression and induction are independent of membrane potential changes. Therefore, AISS’s spikelets express based on voltage-independent sodium channels. In terms of AISS induction, the facilitation of voltage-gated sodium channel (VGSC) activation accelerates AISS onset, or vice versa.

**Conclusion:**

AISS expression in GABAergic neurons is triggered by the spikelets based on the functional emergence of voltage-independent sodium channels, which is driven by intensive VGSCs’ activities.

## Introduction

GABAergic neurons preserve the balance between excitation and inhibition in neuronal networks for the brain to manage well-organized cognition and behavior [1-3]. GABAergic dysfunction presumably leads to disinhibition disorders in the cerebral cortices, such as epilepsy [4-7] and psychopathy [8-11]. The upregulation of GABAergic interneurons is critical to prevent disinhibition-related brain disorders. The function of GABAergic interneurons can be upregulated at three compartments (excitatory synaptic inputs, soma and output synapses). For instance, GABAergic synapses express activity-dependent potentiation [12-15]. Excitatory synapses on GABAergic neurons are potentiated by Ca^2+^ signals [16-18]. The intrinsic properties of GABAergic neurons are changed in behavioral plasticity [19-21]. The persistent spikes in GABAergic neurons are induced by their repetitive activities [22,23].

Activity-induced spontaneous spikes (AISS) at GABAergic neurons are presumably initiated at their axons and modulated by neuropeptides [22,23]. The mechanisms essential for AISS induction and expression remain elusive. As the generation of spikes is function of sodium channels [24-29], we propose to study whether a conversion of evoked spikes into AISS results from a functional emergence of voltage-independent sodium channels and how these sodium channels work for AISS induction and expression.

To address these questions, we conducted the experiments at GABAergic neurons in hippocampal slices. Our strategies include the followings. If the intensive activities of voltage-gated sodium channels (VGSC) drive AISS induction, the facilitation of VGSC activation should shorten induction phase, or vice versa. If AISS expression is associated with a decreased threshold near resting membrane potential, AISS onset is based on the voltage-independence of sodium channels. Furthermore, the functional emergence of such voltage-independent sodium channels for AISS expression will be granted by seeing additional spike capability and two upstroke phases (such as voltage-independent and voltage-dependent sodium currents) in AISS, compared with evoked spikes.

## Results

Electrical signals were recorded at GABAergic neurons that were genetically labeled with GFP in mouse hippocampal slices under current-clamp or voltage-clamp. Depolarization pulses were repetitively injected into the neurons to induce sequential spikes. Some of these neurons were able to fire spontaneous spikes, i.e., activity-induced spontaneous spikes (AISS). We analyzed evoked spikes and AISS, in terms of their threshold potential, spike upstroke, voltage-dependence and pharmacology, in order to figure out mechanisms underlying AISS induction and expression.

### Intensive activity induces spontaneous spikes in hippocampal GABAergic neurons

**Figure 1:**
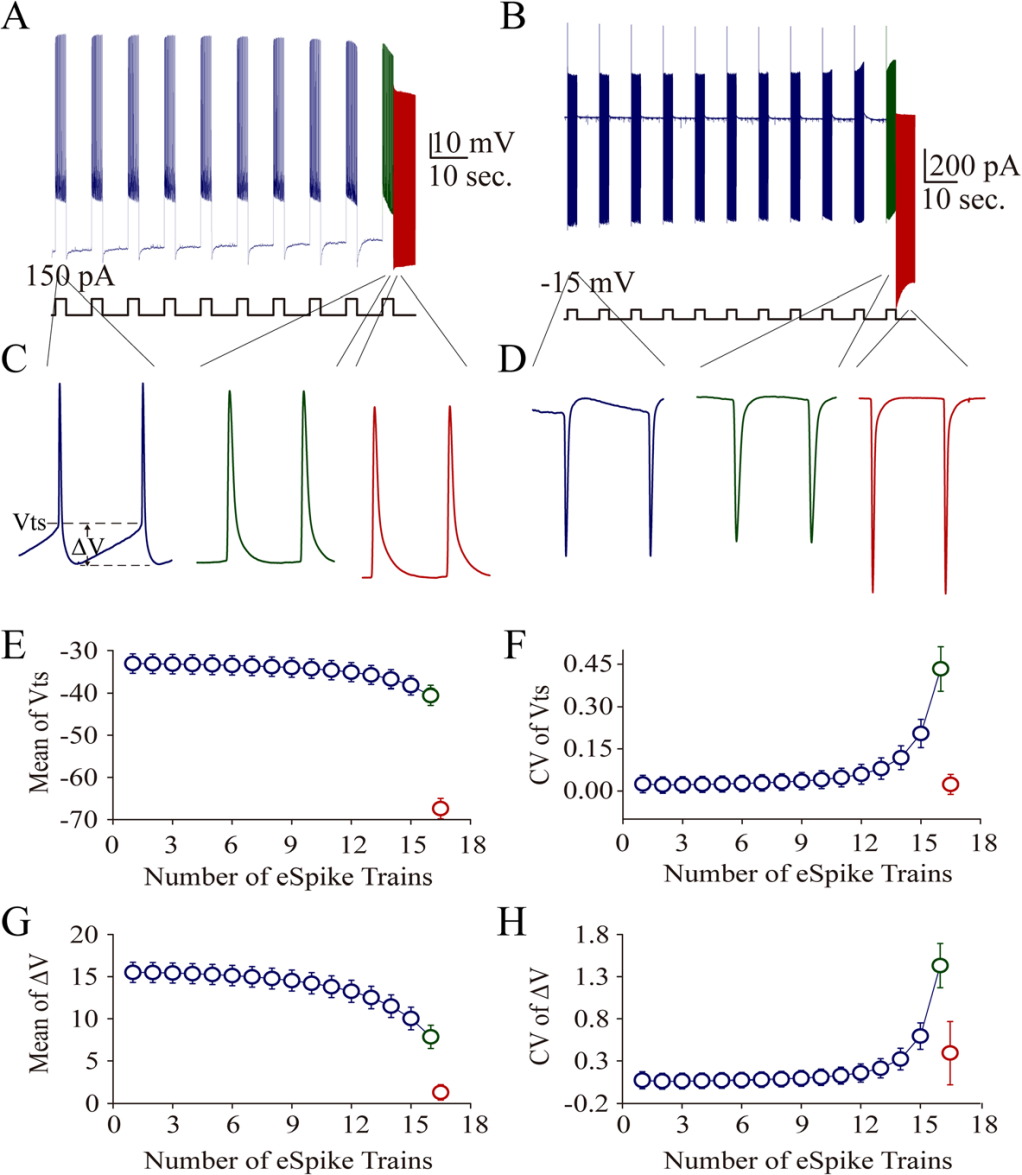
**Intensive activity at hippocampal GABAergic neurons induces spontaneous spikes, i.e., activity-induced spontaneous spikes (AISS), under current-clamp and voltage-clamp.** A sequential depolarization pulses (150 pA or 200 pA/3 seconds with 7 seconds of intervals) were injected into the neurons to evoke the spikes. **A)** shows spikes induced by depolarization currents (150 pA; blue trace) and AISS (red trace) under current-clamp recording. Calibration bars are 10 mV and 10 seconds. **B)** shows spikes induced by depolarization voltages (15 mV; blue trace) and AISS (red trace) under voltage-clamp. Calibration bars are 200 pA and 10 seconds. **C)** illustrates the expanded waveforms of evoked spikes (blue trace), evoked spikes just before AISS (green trace) and AISS (red trace) from panel A. **D)** shows the expanded waveforms of evoked spikes (blue trace), evoked spikes just before AISS (green trace) and AISS (red trace) from panel B. **E)** shows the dynamical change in threshold potential (Vts) during AISS induction. **F)** shows the dynamical change in the coefficient variation (CV) of Vts during AISS induction. **G)** shows the dynamical change in energetic barrier (ΔV) during AISS induction. **H)** shows the dynamical change in the CV of ΔV during AISS induction (n = 23).

Three reports showed that the intensive activity of hippocampal GABAergic interneurons induced spontaneous persistent spikes [22,23,30]. We produced activity-induced spontaneous spikes (AISS) by injecting repetitive depolarization pulses (150 ~ 200 pA/3 seconds; 7 seconds for inter-pulse interval) into hippocampal GABAergic neurons for a few minutes. The conversion of evoked spikes (blue traces) into AISS (reds) is seen under current-clamp (Figure 1A) and voltage-clamp recordings (1B), in which AISS lasts for more than 10 seconds. Six minutes after AISS disappears in these neurons, the depolarization pulses can reproduce AISS (Additional file 1: Figure S1). There is no statistical difference in the number of evoked spikes that are needed for AISS production and reproduction (p > 0.5, Additional file 1: Figure S1). An intensive firing of evoked spikes for AISS expression implies that AISS induction is a spike-driven process. The reproducibility of AISS onset indicates that it is a natural intrinsic property of GABAergic neurons.

Spontaneous spikes can be triggered by spontaneous excitatory synaptic activities and produced based on neuronal intrinsic property. Spike threshold potential can be used as an index to indicate whether AISS onset is synaptic potential dependent or intrinsically voltage-independent. Compared with evoked spikes (blue traces in Figure 1C ~ D) and evoked spikes just before AISS onset (green), AISS waveforms (red traces) show that their threshold potentials (Vts) reduce near the resting membrane potential and their energetic barriers (ΔV; [31] tend to be zero. The values of Vts and ΔV gradually reduce in AISS induction (Figure 1E and 1G, n = 29). Vts is close to the resting membrane potential (red symbols in 1E) and ΔV appears zero just before AISS onset (red symbols in 1G). Therefore, AISS-expressed neurons demonstrate dynamical ΔV decrease. Zero ΔV indicates the voltage-independence of AISS expression. In addition, ΔV decreases dynamically in each evoked spikes train and recovers partially in inter-spike trains, as showed a gradual increase of ΔV coefficient variation (CV) in Figure 1H. The increase of ΔV’s CV in late phase of AISS induction further indicates that the conversion of evoked spikes into AISS expression is a spike-driven process.

In brief, AISS expression is voltage-independent and AISS induction is spike-driven. In terms of AISS expression, we focused on examining whether new voltage-independent sodium channels emerged for AISS expression. If it is a case, we expect to see the decrease of spike threshold potential near resting membrane potential, the inclusion of voltage-independent and voltage-dependent sodium currents in AISS waveforms and the addition of spike ability in AISS above evoked spikes. On the other hand, we studied whether the spike-driven process for AISS induction was based on the intensive activity of voltage-gated sodium channels (VGSC). If it is a case, manipulating VGSC’s dynamics should change the efficiency of AISS induction.

### A voltage-independent component of sodium channel currents emerges for AISS expression

#### AISS expression includes an additional component of spikes beyond evoked spikes

**Figure 2:**
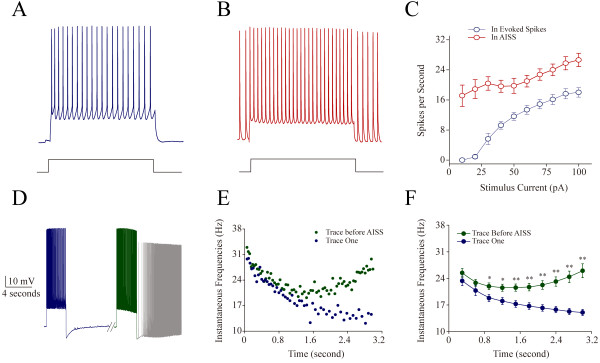
**The capability of firing spikes is higher during AISS expression and induction than the evoked spikes. A** ~ **C)** Input–output curves (depolarization intensities versus spike frequencies) are measured under the conditions of the evoked spikes and subsequent AISS expression in GABAergic neurons. **A)** shows the spikes evoked by a depolarization pulse (100 pA/1 second) at a neuron. **B)** shows the spikes evoked by this depolarization pulse during AISS expression at this neuron. **C)** illustrates spikes per second versus stimulus currents under the conditions of control (blue symbols) and AISS expression (red symbols, n = 8). **D** ~ **E)** Instantaneous spike frequency (1/inter-spike interval) are measured in the spike trains of inducing AISS from the first train and the train just before AISS onset in GABAergic neurons. **D)** shows the spikes evoked by a depolarization pulse (150 pA/3 second) in the first train (blue trace) and the train just before AISS onset (green trace), as well as AISS expression (gray trace) at a neuron. Calibration bars are 10 mV and 4 seconds. **E)** shows instantaneous spike frequency versus time for the spikes induced in the first train (blue symbols) and in the train just before AISS onset (green symbols) at this cell. **F)** Statistical analysis shows instantaneous spike frequency versus time for the spikes induced in the first train (blue symbols) and in the train just before AISS onset (green symbols, asterisks, p < 0.01, n = 29 neurons).

The capability of producing spikes was assessed by analyzing the ability to convert input signals into spikes during AISS versus evoked spikes and by quantifying the change of spike ability just before AISS onset. Input–output curves, an index for showing the ability of signal conversion [26], were measured under the conditions of evoked spikes and AISS. If their input–output curves are similar, the occlusion indicates the sharing of common mechanism. The shift of input–output curve to high level during AISS indicates an addition of new sodium channel currents to evoked spikes. Figure 2A ~ C shows the relationships between spike frequencies versus normalized stimuli under the conditions of evoked spikes and AISS expression. The input–output curve during AISS (red symbols) shifts toward high level, compared to the input–output in evoked spikes (blues; p < 0.01, n = 8). Therefore, there is an additional component of sodium currents for AISS expression beyond those for the evoked spikes.

We confirmed the addition of this new component by analyzing spike capability in evoked spikes train just before AISS onset versus in the first evoked spikes train. If spike frequency is higher in the train just before AISS onset than the first train by a given depolarization pulse, the new component of sodium currents is added into VGSC current for AISS expression. Spike frequency is significantly higher in the train just before AISS onset (green trace and symbols in Figure 2D ~ F) than in the first train (blue trace and symbols; an asterisk, p < 0.05; two asterisks, p < 0.01 n = 29). Thus, a new component of sodium current is added for AISS expression.

The results in Figures 1 and 2 imply an emergence of voltage-independent sodium currents for AISS expression. Moreover, if each of spike waves in AISS is separated into voltage-independent and voltage-dependent components of sodium channel currents that are different in biophysics and pharmacology, this implication will be warranted.

#### The full spikes in AISS include two phases

The hypothesis about adding new voltage-independent component of sodium currents into VGSC ones for AISS expression predicts that spike upstrokes in AISS should be separated into two phases that have different Vts and rising slopes. If the voltage-independent component is somehow unable to trigger voltage-dependent one, we should see the spikelet, or vice versa, such that the spikelets and spikelet-spike mixes are present in AISS. A common approach to merit rising slope and Vts for the spikes is the phase-plots of *dV/dt* versus membrane potentials [32,33]. The conductance, density and activity synchrony of the sodium channels influence the rising slope of the spike upstroke [34,35]. If more than one component is seen in the spike upstrokes, two populations of sodium channels are assumed.

**Figure 3:**
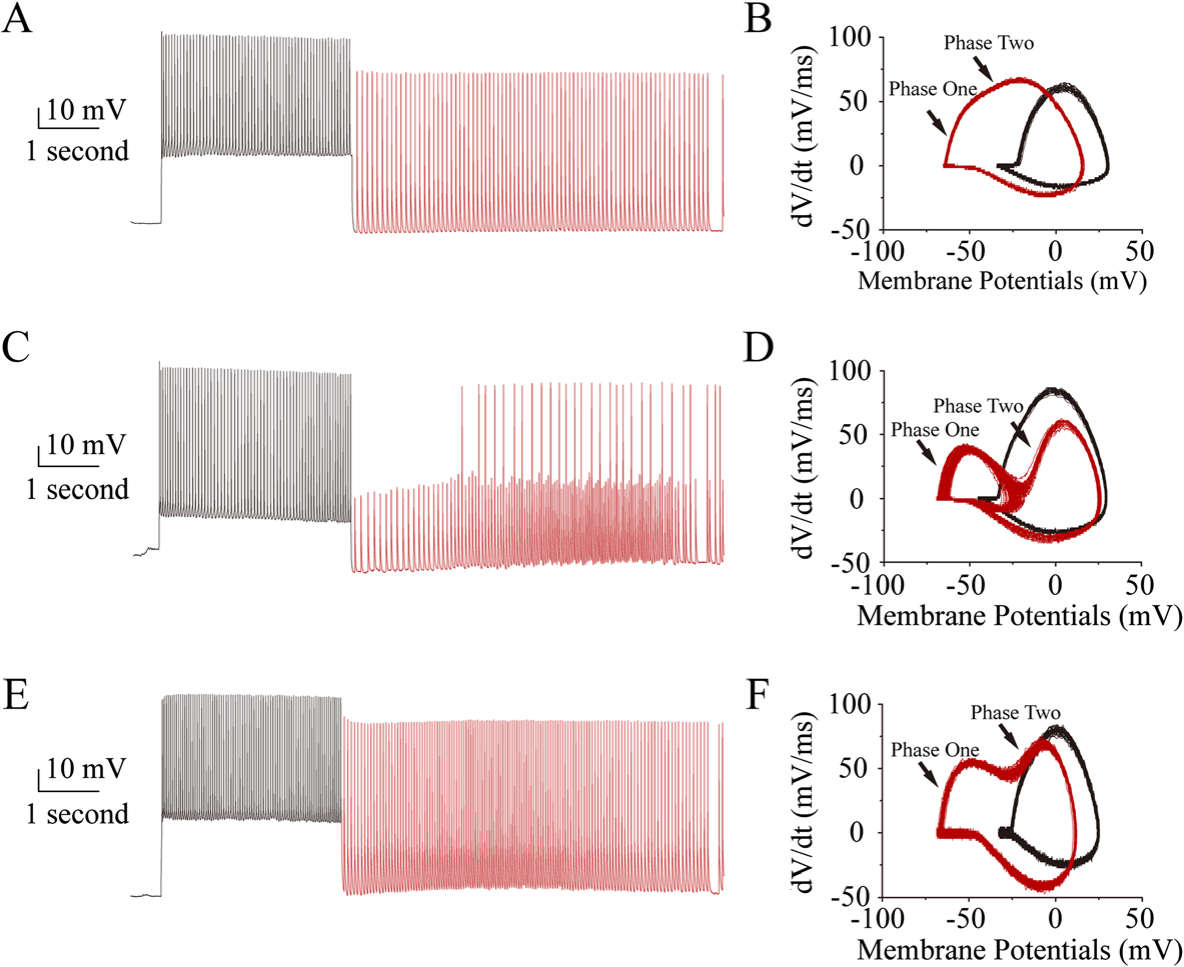
**A single phase of upstroke in the evoked spikes is converted into two phases in AISS expression, in which the rising slopes of phase two are variable compared with phase one. A)** shows AISS onset (red trace) after evoked spikes (black trace) in a GABAergic neuron. **B)** shows the phase-plots for the evoked spikes (black traces) and AISS (red traces) in this neuron. There are two phases in AISS’s rising slopes, phase one and phase two (pointed by arrows). **C)** shows AISS onset (red trace) after evoked spikes (black trace) in a GABAergic neuron, in which AISS includes spikelets and full spikes. **D)** shows the phase-plots for evoked spikes (black traces) and AISS (red traces). In addition to two phases in AISS’s rising slope, there are two formats of AISS for spikelets and full spikes, respectively. **E)** shows AISS onset (red trace) after evoked spikes (black trace) in a GABAergic neuron. **F)** illustrates the phase-plots for evoked spikes (black traces) and AISS (red traces). There is an interval between two phases of AISS. Compared with a single phase for the evoked spikes, all of AISS waveforms show two phases.

Figure 3 shows the comparison in the upstrokes of evoked spikes and AISS under current-clamp recording. Compared with evoked spikes (black traces in Figure 3A, C and E), the upstrokes of AISS (red traces) are divided into two phases based on their rising slopes and onset potentials. There are three types of phase plots for spike waveforms in AISS (red traces in Figure 3B, 3D and 3F), in which phase one is corresponding to the spikelets in AISS and phase two is spikelet-triggered subsequent spikes (Figure 3C). In addition to different Vts values for the spikelets and subsequent spikes, the rising slopes appear larger in phase one than phase two (red traces in Figure 3B, 3D and 3F). These two phases in AISS upstroke are also seen under voltage-clamp recording (Additional file 2: Figure S2). Therefore, compared with the evoked spikes, the spike upstrokes in AISS include phase one in voltage-independent onset and phase two with voltage-dependent initiation, in which voltage-independent component corresponds to the spikelets.

**Figure 4:**
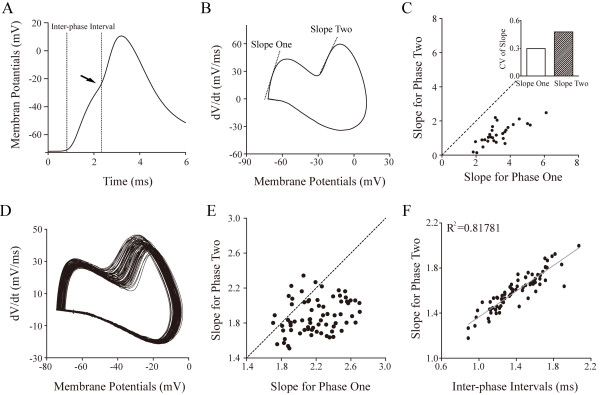
**The rising slopes and threshold potentials in two AISS phases are different, in which phase two is dependent upon phase one. A)** shows an expanded waveform of AISS that has a step-like upstroke with Vts one and two. **B)** shows a phase-plot for this expanded AISS waveform that is divided into two phases with slope one and two. **C)** illustrates a plot of slope two versus slope one from twenty-nine GABAergic neurons. The slope for phase one is larger than that for phase two, and CV of slope two is larger than CV of slope one (inset). There is a linear correlation between slope two and slope one (r^2^ = 0.58). **D)** illustrates phase-plot for the expanded waveforms of AISS that is divided into two phases from a neuron. **E)** shows a plot of slope two versus slope one from this neuron. **F)** illustrates a linear correlation between slope two versus inter-phase intervals (IPI) this neurons, i.e., the onset of phase two is influenced by the refractory period of phase one.

The quantities and relationships for these two phases in terms of their rising slopes are illustrated in Figure 4. The expanded spike wave in AISS appears step-like upstroke (Figure 4A), which is converted to phase-plot (Figure 4B), the rising slopes (20 ~ 80%) for phase one and two versus membrane potentials (Vm). Based on our data, we plot the relationships between phase one slopes and phase two slopes (n = 29 neurons). The absolute slope values are larger for phase one than phase two (Figure 4C), or vice versa in their CV values (inset in 4C). This relationship is also seen in a given neuron (Figure 4D ~ E). In addition, there is a linear correlation between inter-phase intervals and phase two slope (Figure 4F), i.e., component two is influenced by the refractory period of component one. As the phase plot merits the slopes of two components versus Vm, the larger slope values of the spike upstroke (larger *dV/dt* within less Vm change) indicate less voltage-dependence for generating spike upstroke. Therefore, the process of producing phase one is relatively voltage-independent.

#### Spikelets in AISS are independent of membrane potential change compared with full spikes

**Figure 5:**
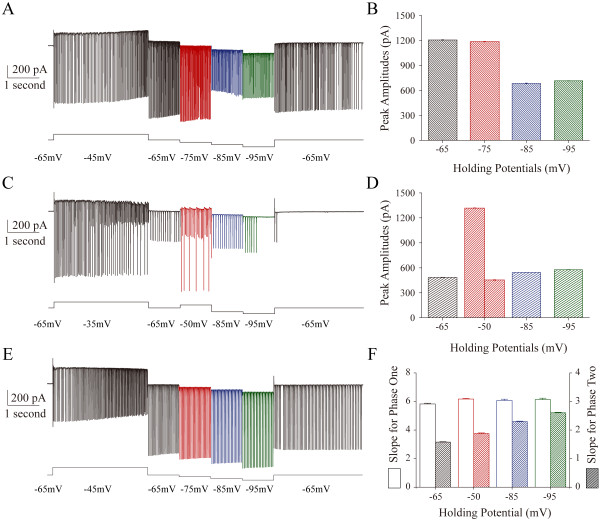
**The conversion between full spikes and spikelets during AISS expression is influenced by the membrane potentials. A)** After AISS onset under voltage-clamp, membrane potentials are hyperpolarized in the steps from −65 mV to −95 mV. A trace shows the spikes evoked just before AISS onset (black trace in the first part) and the AISS under different holding potentials at −65 (black), −75 (red), −85 (blue) and −95 mV (green) in a GABAergic neuron. Full spikes are converted into spikelets at holding potentials of −85 ~ −95 mV. **B)** shows the peak amplitudes of AISS under different membrane potentials of −65 (black), −75 (red), −85 (blue) and −95 mV (green). **C)** A trace illustrates the spikes evoked just before AISS onset (black trace in the first part) and the AISS under different holding potentials at −65 (black), −50 (red), −85 (blue) and −95 mV (green) in a GABAergic neuron. The spikelets are converted into full spikes at holding potential of −50 mV (red trace). **D)** shows the peak amplitudes of AISS under different holding potentials at −65 (black), −50 (red), −85 (blue) and −95 mV (green). **E)** shows the influence of membrane potential on AISS’s phase two, but not phase one. A trace under voltage-clamp recording shows the spikes evoked just before AISS expression (black trace) and the AISS at different holding potentials, −65 (black), −75 (red), −85 (blue) and −95 mV (green) in a GABAergic neuron. **F)** shows the values of slopes for phase one (open bars) and phase two (filled bars) under different holding potentials at −65 (black bar), −75 (red), −85 (blue) and −95 mV (green).

AISS expression was examined under various membrane potentials. After AISS onset under voltage-clamp, we changed membrane potentials in −65 ~ −95 mV. The full spikes in AISS are converted into the spikelets by membrane potential hyperpolarization, and the spikelet amplitudes are independent of hyperpolarization (Figure 5A ~ B). On the other hand, the spikelets in AISS are converted into the full spikes by membrane potential depolarization from −65 to −50 mV (Figure 5C ~ D). Moreover, in AISS phase-plots, the slopes of phase two increase during membrane potential hyperpolarization (filled bars in Figure 5F), but the slopes of phase one do not change (open bars). Therefore, phase two of spike upstroke during AISS expression is affected by membrane potentials, and phase one is insensitive to membrane potentials. AISS expression is voltage-independent.

In summary, full spikes in AISS include two components, i.e., voltage-independent spikelets and voltage-dependent phase two spikes. These spikelets drive membrane potential depolarization toward the threshold that triggers phase two spikes in each of AISS waveforms.

#### The spikelets are less sensitive to TTX than full spikes

**Figure 6:**
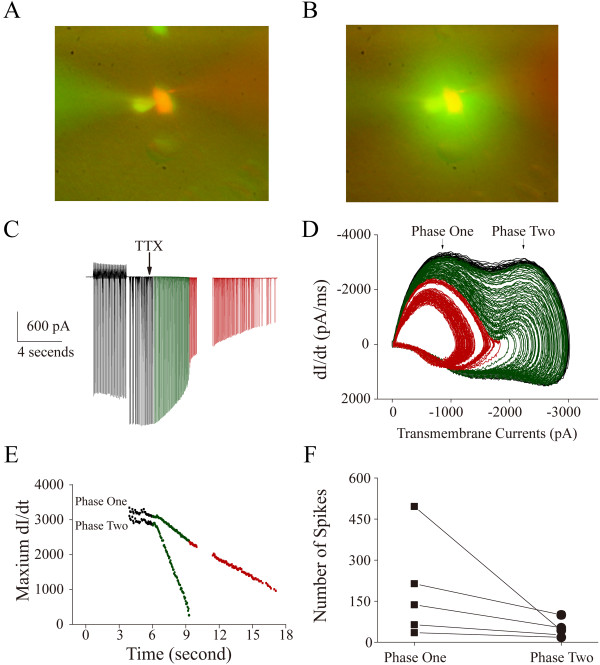
**The sensitivity of AISS phase one and phase two to TTX is different.** TTX (100 nM) is puffed onto GABAergic neurons after AISS onset through a pipette containing Alex-488 (green) in the ACSF. Whole-cell recording pipette contains standard pipette solution (Methods) plus Alex-568 (red fluorescent). **A)** illustrates whole-cell recoding (a red cell) before puffing TTX. **B)** shows whole-cell recording after puffing (green fluorescent around this cell). **C)** shows a trace of the evoked spikes induced just before AISS expression (black trace in the first part) as well as AISS under the control (black trace) and TTX application (green and red traces), in which whole-cell recording is under the voltage-clamp. The spike amplitudes are reduced from full spikes to spikelets by using TTX. **D)** shows the phase-plots for these AISS and spikelet waveforms under the control (black traces) and TTX application (green and red traces). Phase two is completely blocked by TTX (green traces), but phase one is not (reds). **E)** shows dynamical changes in the rising slopes for phase one (green and red symbols) and phase two (green symbols) after TTX application, in which the colors of symbols corresponds to those of traces in panels C ~ D. Phase two is quickly and completely blocked by TTX, but phase one is not. **F)** shows the number of evoked spikes before the blockades of phase one (square symbols, n = 5) and phase two (round symbols).

The sensitivity of phase one and phase two to TTX is showed in Figure 6. The pipettes of recording GABAergic neurons included the pipette solution (Methods) plus 10 μM Alex-568 (red fluorescent in Figure 6A ~ B). The pipettes to puff TTX toward the neurons included the ACSF (Methods) plus 10 μM Alex-488 (green fluorescent in Figure 6A ~ B). After AISS onset in a neuron, TTX application blocks both phase one and two, in which phase two disappears completely in 3 seconds; whereas phase one is not blocked completely in 12 seconds (Figure 6C ~ E). The effects of TTX on phase one and phase two of AISS are seen in other GABAergic neurons (n = 5, Figure 6F).

Compared with phase two, phase one (spikelets) has an onset threshold near to resting membrane potential, its generation independent of membrane potential change and the less sensitivity to TTX. Phase two in AISS and evoked spikes are similar in voltage-dependence, time-dependent recovery (Figure 4F) and TTX sensitivity (Figure 6). Therefore, the generation of the spikelets is based on the newly emerged component of sodium channels that are voltage-independent for AISS expression, but phase two of AISS is controlled by VGSCs.

#### AISS induction is not influenced by membrane potentials

**Figure 7:**
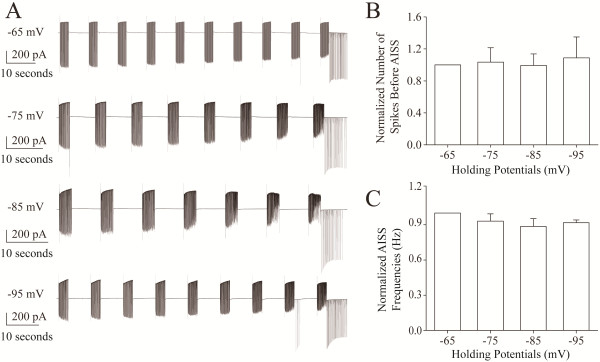
**AISS induction is not affected by membrane potential hyperpolarization.** AISS is induced under the voltage-clamp with the different holding potentials from −65 mV to −95 mV (n = 9). **A)** illustrates AISS induction under different membrane holding potentials at −65 (top trace), −75 (the second trace), −85 (the third trace) and −95 mV (bottom trace) from a GABAergic neuron. Calibration bars are 200 pA and 10 seconds. **B)** shows the number of spikes needed for inducing AISS under the different membrane holding potentials of −65, −75, −85 and −95 mV, in which the values for −75, −85 and −95 mV are normalized by the value for −65 mV. **C)** shows the frequencies of AISS induced under membrane holding potentials at −65, −75, −85 and −95 mV, in which the values for −75, −85 and −95 mV are normalized by the value for −65 mV.

In addition to the voltage-independence of AISS expression, we examined whether membrane potentials influence AISS induction under voltage-clamp recording. Figure 7 illustrates that in −65 ~ −95 mV of membrane potentials, repetitive depolarization pulses induce AISS (Figure 7A) without differences in the need of evoked spikes (Figure 7B) and in the frequency of AISS (Figure 7C). This result is consistent with Figure 5, in which membrane potential hyperpolarization does not remove AISS expression after its onset, but drives a conversion of full spikes into spikelets. That is, AISS expression and induction are voltage-independent. It is noteworthy that less effect of membrane potentials on AISS spikelets (Figures 5 and 7) may also imply the production of the spikelets being away from the somata.

### The activity state of voltage-gated sodium channels influences AISS induction

In terms of AISS induction, we focused on examining a role of voltage-gated ion channels based on the following facts. AISS was induced by membrane depolarization without synaptic stimulation in the GABAergic neurons. AISS induction was a spike-driven process. Because of the influence of potassium channels on sequential spikes, a role of voltage-gated potassium channel in AISS induction was examined by applying tetraethylammonium (TEA). AISS was induced in the presence of 40 mM TEA (Additional file 3: Figure S3), in which the blockade of potassium channels was demonstrated by an incomplete repolarization (arrow). Because of the low threshold for T-type calcium channels, similar to AISS onset, a role of T-type calcium channel in AISS induction was examined by using its inhibitor mibefradil. AISS was induced in the presence of 100 μM mibefradil (Additional file 4: Figure S4). Such results indicate that the activity state of VGSCs may be involved in the spike-driven process for AISS induction.

To examine a role of VGSCs in AISS induction, we observed whether the upregulation of VGSC dynamics accelerated AISS induction or the downregulation of VGSC dynamics postponed its induction. VGSC dynamics was upregulated by using ATX-II (a blocker of VGSC inactivation; [35,36] or hyperpolarization [37], but was downregulated by using anandamide [38]. In the following experiments, we induced AISS in control ACSF. After AISS disappeared for six minutes, we re-induced AISS in one of these pharmacological reagents or hyperpolarization, a protocol similar to Additional file 1: Figure S1.

**Figure 8:**
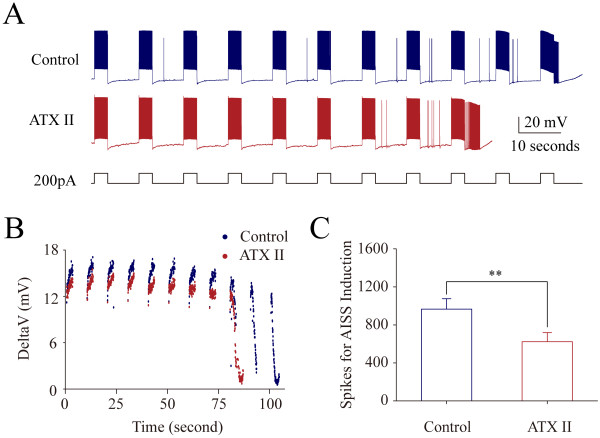
**ATX-II accelerates AISS induction at hippocampal GABAergic neurons.** AISS is induced under the control and subsequent application of ATX-II (50 nM) after the first round of AISS disappears for 6 minutes, a time interval similar to Figure 1. **A)** illustrates AISS induction under the control (top trace) and in presence of ATX-II (bottom trace). Calibration bars are 20 mV and 10 seconds. **B)** shows dynamical changes in threshold potentials under the control (blue symbols) and in presence of ATX-II (red symbols). **C)** shows the number of spikes needed for AISS onset under the control (blue bar) and in presence of ATX-II (red bar; asterisk, p < 0.05, n = 8).

**Figure 9:**
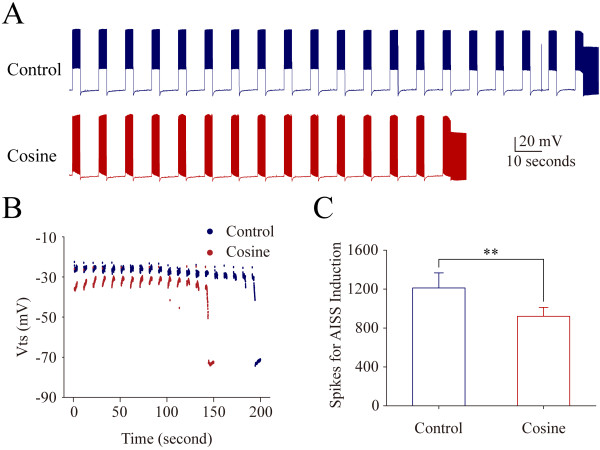
**Depolarization pulses in the pattern of cosine waves accelerate AISS induction at hippocampal GABAergic neurons.** Cosine waveforms include depolarization and hyperpolarization. AISS is induced under the control and subsequent cosine waveforms after the first round of AISS disappears for 6 minutes, a time interval similar to Figure 1. **A)** shows AISS induction under the control (top trace) and the cosine (Cos) waveforms (bottom trace). Calibration bars are 20 mV and 10 seconds. **B)** illustrates the dynamical changes in threshold potentials under the control (blue symbols) and cosine waveforms (red symbols). **C)** shows the number of spikes needed for AISS onset under the control (blue bar) and cosine waveforms (red bar; asterisk, p < 0.01, n = 8).

**Figure 10:**
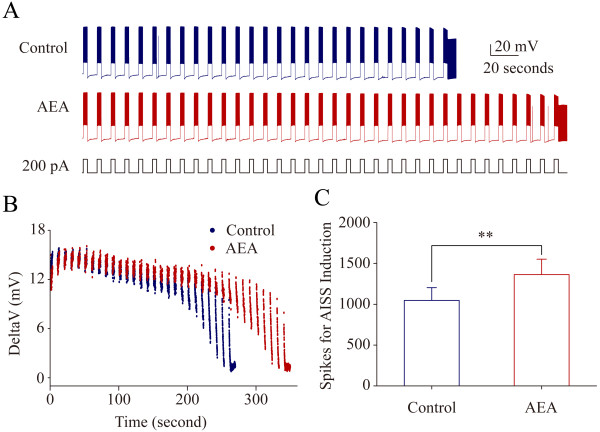
**Anandamide (AEA) postpones AISS induction at hippocampal GABAergic neurons.** AISS is induced under the control and subsequent use of AEA (5 μM)I after the first round of AISS disappears for 6 minutes, a time interval similar to Figure 1. **A)** shows AISS induction under the control (top trace) and in presence of AEA (bottom trace). Calibration bars are 20 mV and 10 seconds. **B)** shows the dynamical changes in threshold potentials under the control (blue symbols) and in presence of AEA (red symbols). **C)** illustrates the number of spikes needed for AISS onset under the control (blue bar) and in presence of AEA (red bar; asterisk, p < 0.01, n = 9).

The upregulation of VGSC function accelerates AISS induction. 50 nM ATX reduces the number of evoked spikes needed to induce AISS (p = 0.034, n = 8; Figure 8B ~ C). Moreover, the cosine waveforms (the combined depolarization and hyperpolarization pulses) reduce the number of evoked spikes needed to induce AISS (p = 0.002, n = 8; Figure 9). On the other hand, the downregulation of VGSC dynamics can postpone AISS induction. 5 μM anandamide increases the number of the evoked spikes needed for AISS induction (p = 0.003, n = 9; Figure 10). The data indicate that the functional status of VGSCs is essential for AISS induction, consistent with an indication of the spike-driven or activity-dependent process for AISS induction.

## Discussion

Intensive activity in hippocampal GABAergic neurons induces spontaneous spikes (Figure 1). An onset of the activity-induced spontaneous spike (AISS) is accelerated by VGSC activation and delayed by VGSC inactivation (Figures 8, 9 and 10). AISS induction is a spike-driven process based on intensive activities of VGSC. After AISS expression, the upstrokes of full spikes in AISS are separated into a newly emerged voltage-independent component of sodium currents in early phase (i.e., spikelet) and a voltage-dependent component in late phase (Figures 2, 3 and 4). This early phase is featured as a zero threshold potential and TTX sensitivity in its expression and voltage-independence in its induction (Figures 3, 4, 5, 6 and 7). AISS production is based on the functional emergence of voltage-independent sodium channels in neuronal processes driven by intensive VGSC-mediated spiking.

In terms of the physiological impact of AISS expression in GABAergic neurons, AISS onset may be self-compensation processes in their networks and individual neurons. Intensive activities in inhibitory neurons hint an over-excited state of their local network. AISS onset helps to suppress this overexcitation in these network neurons. Moreover, once AISS onset at a GABAergic neuron, an emergence of voltage-independent sodium channels is associated with an attenuation of VGSC’s activity (Figures 2, 3, and 4). These functionally attenuated VGSCs can still be activated by the spikelets mediated by newly emerged voltage-independent sodium channels. A mutual substitution in the functions of VGSCs and voltage-independent sodium channels is a basis that AISS’s onset compensates the weakness of evoked spikes (Figure 2D ~ E). These facts grant a notion that the neural homeostasis is maintained among subcellular compartments and network neurons [16].

The induction of AISS at GABAergic neurons is a spike-driven process that the sufficient amount of evoked spikes is needed (Figure 1; also see [22,23]). Compared to the control, a facilitation of VGSC activation reduces the number of spikes needed for AISS induction (Figure 8 and 9), and an attenuation of VGSC activities requires more spikes for AISS induction (Figure 10). Therefore, the functional states of VGSCs control AISS induction. The processes underlying VGSC-driven AISS onset remain elusive. Previous studies show that the gap junctions and neuropeptides modulate AISS induction [22,23]. How the multiple processes coordinately regulate AISS induction needs to be addressed. It is noteworthy that the voltage-dependent potassium channels may not be required for AISS induction (Additional file 3: Figure S3), however, they may be involved AISS expression since the shape of AISS repolarization changes (Figure 1C ~ D), which will be examined in our future study.

In the analysis of AISS waveforms by phase-plot, two phases are distinguished in their upstrokes based on biophysical and pharmacological properties. AISS’s early phase shows zero threshold potential, faster upstroke, voltage-independence and less TTX-sensitivity, compared with its late phase (Figures 3, 4, 5 and 6). This early phase triggers the late phase. The features of the early phase (spikelets) indicate the functional emergence of voltage-independent sodium channels for the AISS expression, which are functionally silent when hippocampal GABAergic neurons are not quite active. It remains to be investigated the mechanisms underlying the turning-on of voltage-independent sodium channels through their structural changes and/or intracellular molecular modulation.

The location of AISS onset was presumably axonal in origin because AISS was induced under the condition of axonal stimuli and somatic hyperpolarization [23]. In our study, AISS can be induced when membrane potentials are hyperpolarized (Figure 7). The expression of AISS’s early phase (spikelets) are not influenced by the hyperpolarization (Figures 5, 6 and 7). A suggestion of axonal origin for the onset of AISS spikelets is strengthened. As AISS’s late phase is influenced by membrane potential, i.e., somatic in origin, the axonal spikelets need to be propagated to the somata to trigger the second phase of individual spikes in AISS. In these regards, the emergence of voltage-independent sodium channels for AISS expression may not be converted from the functional upregulation of somatic VGSCs, but is likely from the turn-on of axonal voltage-independent sodium channels that are silent in inactive neurons.

In terms of the molecular events from the intensive activation of somatic VGSCs to the functional emergence of axonal voltage-independent sodium channels for AISS onset, our study by calcium imaging shows the rise of intracellular Ca^2+^ levels during AISS induction (Additional file 5: Figure S5, n = 3). However, the preloading of 1 mM BAPTA intracellularly by the recording pipettes does not prevent AISS induction and expression (Additional file 5: Figure S5, n = 3). A rise of intracellular Ca^2+^ level is not required for triggering an emergence of voltage-independent sodium channels to induce AISS expression. Since AISS is a spike-driven process, other ions related to fire intensive spikes should be considered to be the triggers for AISS induction. Moreover, the persistent time from AISS onset to disappearance is often less than one minute (Figure 1, 2 and 3), this short-term reversible process may present either the clearance of intracellular accumulated ions or the dynamic activation-to-inactivation of certain enzymes during AISS expression. In terms of ions and enzymes, our testable hypothesis is that sodium and its regulated proteins are responsible for turning on silent voltage-independent sodium channels.

One could question why the increase of intracellular Ca^2+^ is not required for AISS induction. We interpret that an increased Ca^2+^ activates protein kinases, such as PKC and CaM-KII, which downregulate VGSCs’ activity [16,39-43]. This downregulation of VGSC functions during AISS expression leads to the decrease of VGSC-dependent late phase (Figure 4). Another issue is that the results about AISS production are different from lowering intracellular Ca^2+^ level by BAPTA (Additional file 5: Figure S5) and manipulating extracellular Ca^2+^ concentration [23]. Our interpretations to this question are given below. The extracellular Ca^2+^ may affect entire neural networks including excitatory and inhibitory neurons, and in turn multiple neurons influence GABAergic neurons expressing AISS. Moreover, intracellular Ca^2+^ level is influenced by calcium entrance from extracellular space and calcium release from intracellular stores, i.e., cytoplasm Ca^2+^ is not solely from extracellular space. In other words, the results from manipulating intracellular Ca^2+^ and altering extracellular Ca^2+^ level are not comparable.

**Figure 11:**
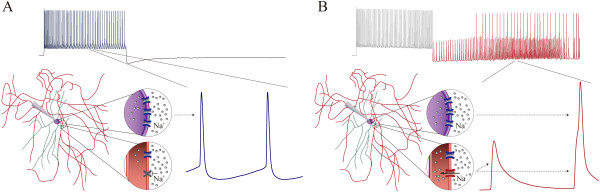
**A diagram shows the conversion of silent voltage-independent sodium channel into active one in the axon of a GABAergic neuron after AISS expression. A)** illustrates the functional silence of voltage-independent sodium channels (gray) on the axon, whereas VGSCs (blue) are active for the generation of the evoked spikes. **B)** illustrates the active state of this voltage-independent sodium channels on the axon (red), which are responsible for generating the spikelets or early phase of full spikes in AISS. VGSCs (blue) are activated for late phase of full spikes in AISS.

In summary, intensive activities in hippocampal GABAergic neurons induce spontaneous spikes. The induction of this activity-induced spontaneous spike, abbreviated as AISS, is a somatic spike-driven and VGSC-mediated process. AISS expression is based on the conversion of axonal voltage-independent sodium channels into active ones. Figure 11 illustrates a hypothetical diagram in the sequence of intensive VGSC activity, voltage-independent sodium channel’s emergence and AISS expression.

## Methods and materials

### Brain slices

Procedures for cutting brain slices and doing electrophysiological experiments were approved by the Institutional Animal Care and Use Committee in the Administration Office of Laboratory Animals in Beijing China (B10831). Hippocampal slices (300 μm) were prepared from C57(GAD67-GFP) mice whose GABAergic neurons were genetically labeled by the green fluorescent protein (gifted by Dr. Yuchio Yanagawa, Gunma University Graduate School of Medicine, Japan; [44-46]. Mice in postnatal days 18–22 were anesthetized by injecting chloral hydrate (300 mg/kg) and decapitated by a guillotine. The slices were sectioned with Vibratome in the oxygenized (95% O_2_/5% CO_2_) artificial cerebrospinal fluid (mM: 124 NaCl, 3 KCl, 1.2 NaH_2_PO_4_, 26 NaHCO_3_, 0.5 CaCl_2_, 5 MgSO_4_, 10 dextrose and 5 HEPES; pH 7.35) at 4°C, and subsequently were held in normal oxygenated ACSF (mM: 124 NaCl, 3 KCl, 1.2 NaH_2_PO_4_, 26 NaHCO_3_, 2.4 CaCl_2_, 1.3 MgSO_4_, 10 dextrose and 5 HEPES; pH 7.35) 24°C for 1 ~ 2 hours before the experiments. A slice was placed to a submersion chamber (Warner RC-26G) that was perfused by the normal ACSF at 31°C for whole-cell recordings [10,20,47-50].

### Electrophysiological study in hippocampal GABAergic neurons

A selection of hippocampal GABAergic neurons for whole-cell recording was based on the following criteria. These neurons in CA1 area of hippocampal stratum radiatum appeared smaller round soma and multiple processes, compared to relatively larger pyramidal neurons in pyramidale, under the DIC microscope (Nikon, FN-E600). These neurons labeled by GFP were identified under fluorescent microscope (Nikon, FN-E600). They fired fast sequential spikes with less adaptation in amplitudes and frequency, typical properties for the interneurons [1-3,51-55].

These interneurons were recorded by an amplifier (MultiClapm-700B, Axon Instrument Inc, CA USA) under whole-cell current-clamp and voltage-clamp. Electrical signals were inputted into pClamp-10 (Axon Instrument Inc) with a sampling rate at 20 kHz. Transient capacitance was compensated and output bandwidth was 3 kHz. Pipette solution for recording action potentials included (mM) 150 K-gluconate, 5 NaCl, 0.4 EGTA, 4 Mg-ATP, 0.5 Tris- GTP, 4 Na-phosphocreatine and 5 HEPES (pH 7.4 adjusted by 2 M KOH). The osmolarity of pipette solution made freshly was 295–305 mOsmol. The pipette resistance was 8 ~ 10 MΩ.

The action potentials were recorded under the conditions of the current-clamp and voltage-clamp. Activity-induced spontaneous spikes (AISS) were driven by injecting depolarization pulses (200 pA and 3 seconds) in a pattern of direct current (DC) with inter-pulse intervals in 7 seconds. The strength of these depolarization pulses was sufficient to evoke the sequential spikes. The periods of injecting depolarization pulses were given until seeing AISS. The period of AISS expression lasted for a range of 10 ~ 30 seconds. After AISS disappeared for 6 minutes, we re-injected these depolarization pulses to induce AISS in each GABAergic neuron under the conditions of DC, DC plus pharmacological reagents and cosine wave. The protocols were used to examine the effects of different manipulations on the efficiency of AISS induction.

The spikes including the evoked and spontaneous ones in our study were analyzed in terms of the threshold potential (membrane potential for spike onset, abbreviation as Vts; Figure 1C), energetic barrier (a difference between threshold potential and resting membrane potential, abbreviation as ΔV; [31] and Figure 1C), spike frequency (Figure 2), spike phase-plot (spike dV/dt vs. membrane potential, Figure 3), input–output (current input versus spike output in Figure 2A ~ C) and spike voltage-dependence (spikes versus membrane holding potentials in Figures 5 and 7).

Spike threshold potentials and ΔV can be used to evaluate whether the spikes are easily induced and to denote how voltage-gated sodium channels (VGSC) are activated [16,26,31,34,37,56]. After AISS onset, the spikes are separated into two phases in GABAergic neurons (Figures 2 and 3). The threshold potential for the spikelets is a membrane potential for their onset, and the threshold potential for subsequent spikes is a membrane potential for the onset of the second phase spikes (Figure 4). As the dV/dt values of spike’s rising phases reflect the efficiencies of individual VGSC activation and of multiple VGSCs’ activation [57,58], the positive dV/dt values in spike phase-plots indicate the efficiency of synchronous activation in a population of VGSCs. The spike frequency and input–output curve will indicate the neuronal ability to convert input signals into digital spikes as well as the addition of new components into spike rising phase [26,47]. Spontaneous spikes versus membrane holding potentials are used to test whether membrane potentials affect the induction and expression of AISS as well as the onset of AISS phases. These analyses were done under the conditions of DC (control) and various manipulations (biophysics and pharmacology), so that we are able to identify whether the functional states of sodium currents are associated with the onset of AISS.

The evoking of sequential spikes by DC and cosine waves was based on a fact that the waves to drive spike generation *in vivo* were classified as steady-state and fluctuated formats [35,47], in which the cosine waveforms presumably allowed VGSCs’ recovery from previous inactivation. In the analyses of input–output curves [26], the depolarization pulses (1 second) in various intensities were injected into the interneurons to induce sequential spikes before and after AISS onsets. This analysis indicates the ability of these interneurons to convert excitatory inputs into spikes and the probability of VGSCs’ activation by excitatory inputs i.e., neuronal sensitivity to the excitatory inputs [16,31,34,56].

It is noteworthy that we measure upstroke velocity in two phases of AISS’s spikes, respectively, instead of other properties, such as spike durations at half-height and spike amplitudes. dV/dt values well represent VGSCs’ efficiency in their synchronous activation [59]. However, as two rising phases of AISS spikes are present, the spike duration at half-height includes the mixture of the two components, i.e., this parameter is influenced by two separate mechanisms. Moreover, spike amplitudes are affected by VGSCs and electrochemical gradients. In these regards, spike duration and amplitude may not be optimal to characterize the mechanisms of voltage-dependent and voltage-independent VGSCs in AISS onset.

The data were analyzed if the recorded neurons had resting membrane potentials negatively more than −60 mV and action potentials above 90 mV. The criteria for the acceptation of each experiment also included less than 5% changes in resting membrane potential, spike magnitudes, input and seal resistances throughout each experiment. The values for all parameters are presented as mean ± SE. The comparisons among groups are done in one way ANOVA, and before versus after treatment are done with paired t-test [60].

## Competing interests

All authors declare that they have no competing interests.

## Authors’ contribution

WL, BW and FZ contribute to experiments and data analyses. JHW contributes to project design and paper writing. All authors have read and approved final version.

## Supplementary Material

Additional file 1: Figure S1 The intensive activities at the hippocampal GABAergic neurons induce spontaneous spikes, activity-induced spontaneous spikes (AISS), which is reproducible. **A)** illustrates the sequential induction of AISS, in which AISS can be induced after previous AISS disappears for 6 minutes. Calibration bars are 10 mV/10 seconds. **B)** shows the number of evoked-spike traces needed for inducing AISS in the first round and the second round (n = 23). **C)** illustrates the number of evoked spikes needed for inducing AISS in the first round and the second round (n = 23). Click here for file

Additional file 2: Figure S2 A single phase of upstroke in evoked spikes is converted into two phases in AISS expression under voltage-clamp recording, in which the rising slopes of phase two are variable compared with phase one. **A)** illustrates AISS expression (red trace) after evoked spikes (black trace) in a GABAergic neuron. **B)** shows phase-plots for evoked spikes (black traces) and AISS (red traces) in this neuron. There are two phases in AISS’s rising slopes, phase one and phase two (pointed by arrows). **C)** shows AISS onset (red trace) after evoked spikes (black trace) in a GABAergic neuron, in which AISS includes spikelets and full spikes. **D)** shows the phase-plots for evoked spikes (black traces) and AISS (red traces). In addition to two phases in AISS’s rising slope, there are two formats of AISS for spikelets and full spikes, respectively. **E)** illustrates AISS onset (red trace) after evoked spikes (black trace) in a GABAergic neuron. **F)** illustrates the phase-plots for evoked spikes (black traces) and AISS (red traces). There is an interval between two phases of AISS. Compared with a single phase for the evoked spikes, all of AISS waveforms show two phases. Click here for file

Additional file 3: Figure S3 AISS induction in GABAergic neurons does not require voltage-gated potassium channels. **A)** AISS is induced by their intensive activity under the control condition. **B)** After AISS disappears for six minutes, their intensive activity induces AISS in the presence of 40 mM TEA, a blocker of voltage-gated potassium channels. TEA effectiveness can be sure by observing the incomplete repolarization (indicated by arrow). Calibration bars are 10 mV and 1 second. Click here for file

Additional file 4: Figure S4 AISS induction in GABAergic neurons does not require voltage-gated low threshold calcium channels. **A)** AISS is induced by their intensive activity under the control. **B)** After AISS disappears for six minutes, their intensive activity induces AISS in presence of 100 μM mibefradil, a blocker of voltage-gated calcium channels that possess low threshold. Calibration bars are 10 mV and 1 second. Click here for file

Additional file 5: Figure S5 AISS induction and expression are accompanied by the intracellular Ca^2+^ elevation in hippocampal GABAergic neurons, but blocked by preloading 1 mM BAPTA in these neurons. **A)** The level of intracellular Ca^2+^ was measured by loading fluo-3 into the recorded neurons through the recording pipette and by quantifying the fluorescent intensity under a laser scanning confocal microscope. Top panel shows the dynamic changes of fluorescent intensity in that the level of intracellular Ca^2+^ based on the binding of fluo-3 with Ca^2+^ is proportional to the spike trainings for evoking AISS. Bottom panel shows the process of AISS induction and expression. **B)** The intracellular preloading of BAPTA does not prevent AISS induction and expression. 1 mM BAPTA was included in the recording pipette. After the formation of whole-cell recording for 5 minutes, the depolarization pulses were injected into the neurons to induce AISS. The use of 1 mM BAPTA is based on the following considerations. 1) In general, the intracellular Ca^2+^ increases to 10^-4~-5^ during cell activation from 10^-8^ under the resting condition, and one BAPTA is able to bind two Ca^2+^ so that 1 mM BAPTA in the recording neurons should be able to buffer Ca^2+^ increase from intensive neuron activity. 2) This concentration of BAPTA was sufficient to block the functional plasticity induced in the neurons. 3) BAPTA is an acidic reagent. The high concentration of BAPTA in the recording pipettes may induce cellular acidosis. The high concentration of BAPTA will change the osmolarity of the recording neurons. This condition leads to the study not being physiological in nature. Click here for file
