# Supplementary material for: Voltage-independent sodium channels emerge for an expression of activity-induced spontaneous spikes in GABAergic neurons
Source: Mol Brain. 2014 May 20;7:38. doi: 10.1186/1756-6606-7-38 (PMC4039334; doi:10.1186/1756-6606-7-38)
Supplement: Additional file 1: Figure S1 — The intensive activities at the hippocampal GABAergic neurons induce spontaneous spikes, activity-induced spontaneous spikes (AISS), which is reproducible. A) illustrates the sequential induction of AISS, in which AISS can be induced after previous AISS disappears for 6 minutes. Calibration bars are 10 mV/10 seconds. B) shows the number of evoked-spike traces needed for inducing AISS in the first round and the second round (n = 23). C) illustrates the number of evoked spikes needed for inducing AISS in the first round and the second round (n = 23). [file 1756-6606-7-38-S1.doc]

**
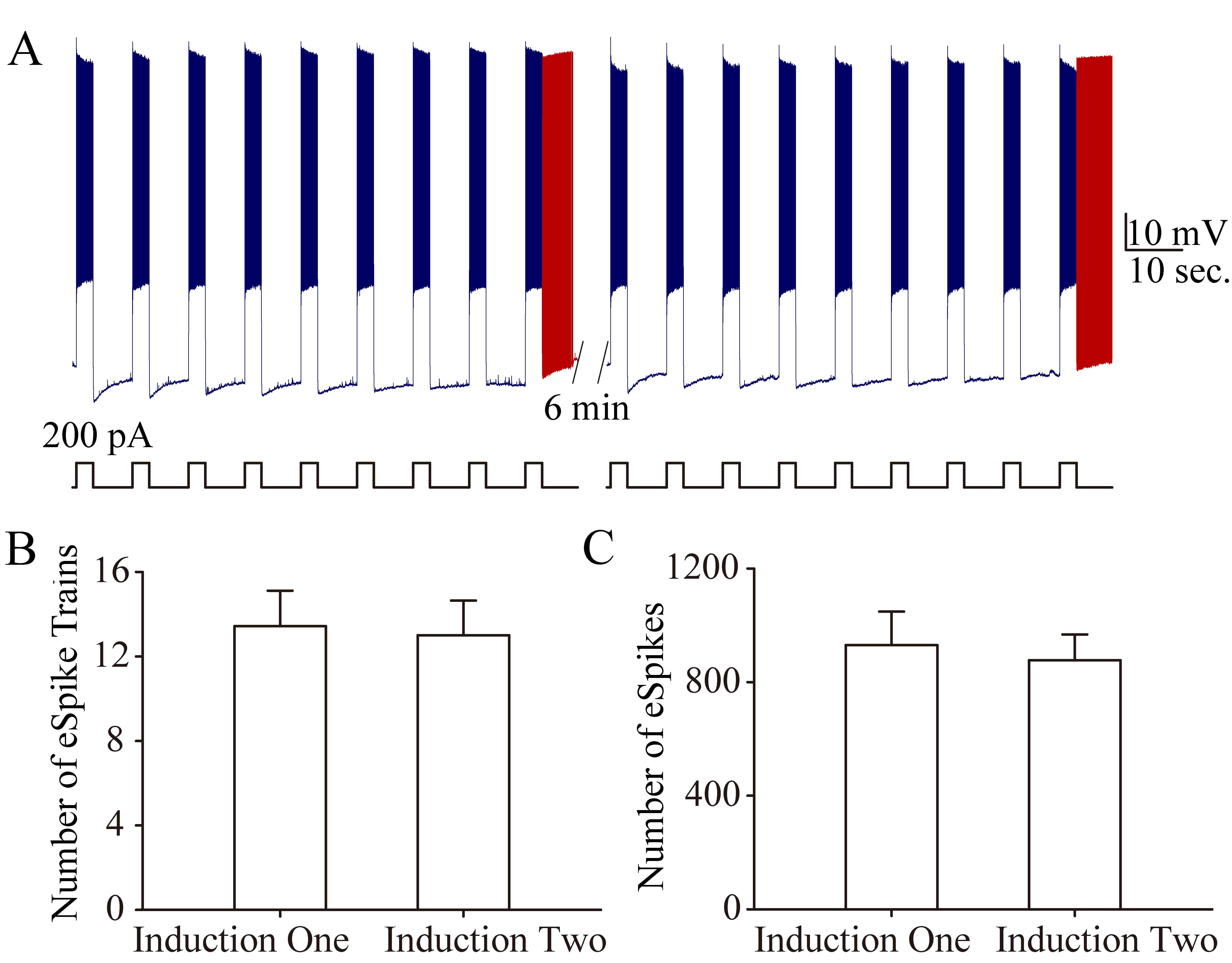
**

**Additional file one: Figure S1** The intensive activities at the hippocampal GABAergic neurons induce spontaneous spikes, activity-induced spontaneous spikes (AISS), which is reproducible. **A)** illustrates the sequential induction of AISS, in which AISS can be induced after previous AISS disappears for 6 minutes. Calibration bars are 10 mV/10 seconds. **B)** shows the number of evoked-spike traces needed for inducing AISS in the first round and the second round (n=23). **C)** illustrates the number of evoked spikes needed for inducing AISS in the first round and the second round (n=23).
