# Supplementary material for: Voltage-independent sodium channels emerge for an expression of activity-induced spontaneous spikes in GABAergic neurons
Source: Mol Brain. 2014 May 20;7:38. doi: 10.1186/1756-6606-7-38 (PMC4039334; doi:10.1186/1756-6606-7-38)
Supplement: Additional file 2: Figure S2 — A single phase of upstroke in evoked spikes is converted into two phases in AISS expression under voltage-clamp recording, in which the rising slopes of phase two are variable compared with phase one. A) illustrates AISS expression (red trace) after evoked spikes (black trace) in a GABAergic neuron. B) shows phase-plots for evoked spikes (black traces) and AISS (red traces) in this neuron. There are two phases in AISS’s rising slopes, phase one and phase two (pointed by arrows). C) shows AISS onset (red trace) after evoked spikes (black trace) in a GABAergic neuron, in which AISS includes spikelets and full spikes. D) shows the phase-plots for evoked spikes (black traces) and AISS (red traces). In addition to two phases in AISS’s rising slope, there are two formats of AISS for spikelets and full spikes, respectively. E) illustrates AISS onset (red trace) after evoked spikes (black trace) in a GABAergic neuron. F) illustrates the phase-plots for evoked spikes (black traces) and AISS (red traces). There is an interval between two phases of AISS. Compared with a single phase for the evoked spikes, all of AISS waveforms show two phases. [file 1756-6606-7-38-S2.doc]

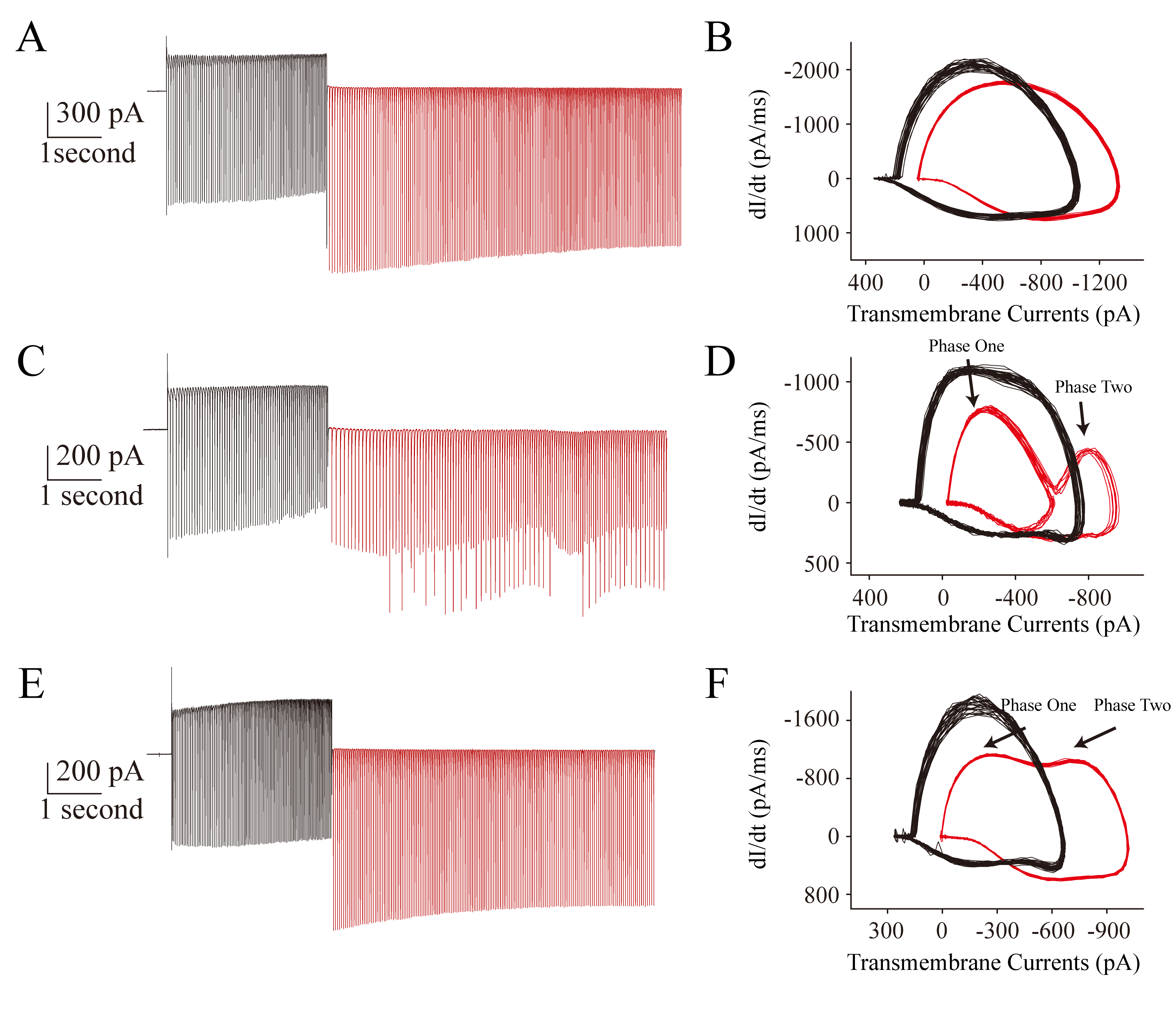


**Additional file two: Figure S2** A single phase of upstroke in evoked spikes is converted into two phases in AISS expression under voltage-clamp recording, in which the rising slopes of phase two are variable compared with phase one. **A)** illustrates AISS expression (red trace) after evoked spikes (black trace) in a GABAergic neuron. **B)** shows phase-plots for evoked spikes (black traces) and AISS (red traces) in this neuron. There are two phases in AISS’s rising slopes, phase one and phase two (pointed by arrows). **C)** shows AISS onset (red trace) after evoked spikes (black trace) in a GABAergic neuron, in which AISS includes spikelets and full spikes. **D)** shows the phase-plots for evoked spikes (black traces) and AISS (red traces). In addition to two phases in AISS’s rising slope, there are two formats of AISS for spikelets and full spikes, respectively. **E)** illustrates AISS onset (red trace) after evoked spikes (black trace) in a GABAergic neuron. **F)** illustrates the phase-plots for evoked spikes (black traces) and AISS (red traces). There is an interval between two phases of AISS. Compared with a single phase for the evoked spikes, all of AISS waveforms show two phases.
