# Supplementary material for: Voltage-independent sodium channels emerge for an expression of activity-induced spontaneous spikes in GABAergic neurons
Source: Mol Brain. 2014 May 20;7:38. doi: 10.1186/1756-6606-7-38 (PMC4039334; doi:10.1186/1756-6606-7-38)
Supplement: Additional file 3: Figure S3 — AISS induction in GABAergic neurons does not require voltage-gated potassium channels. A) AISS is induced by their intensive activity under the control condition. B) After AISS disappears for six minutes, their intensive activity induces AISS in the presence of 40 mM TEA, a blocker of voltage-gated potassium channels. TEA effectiveness can be sure by observing the incomplete repolarization (indicated by arrow). Calibration bars are 10 mV and 1 second. [file 1756-6606-7-38-S3.doc]

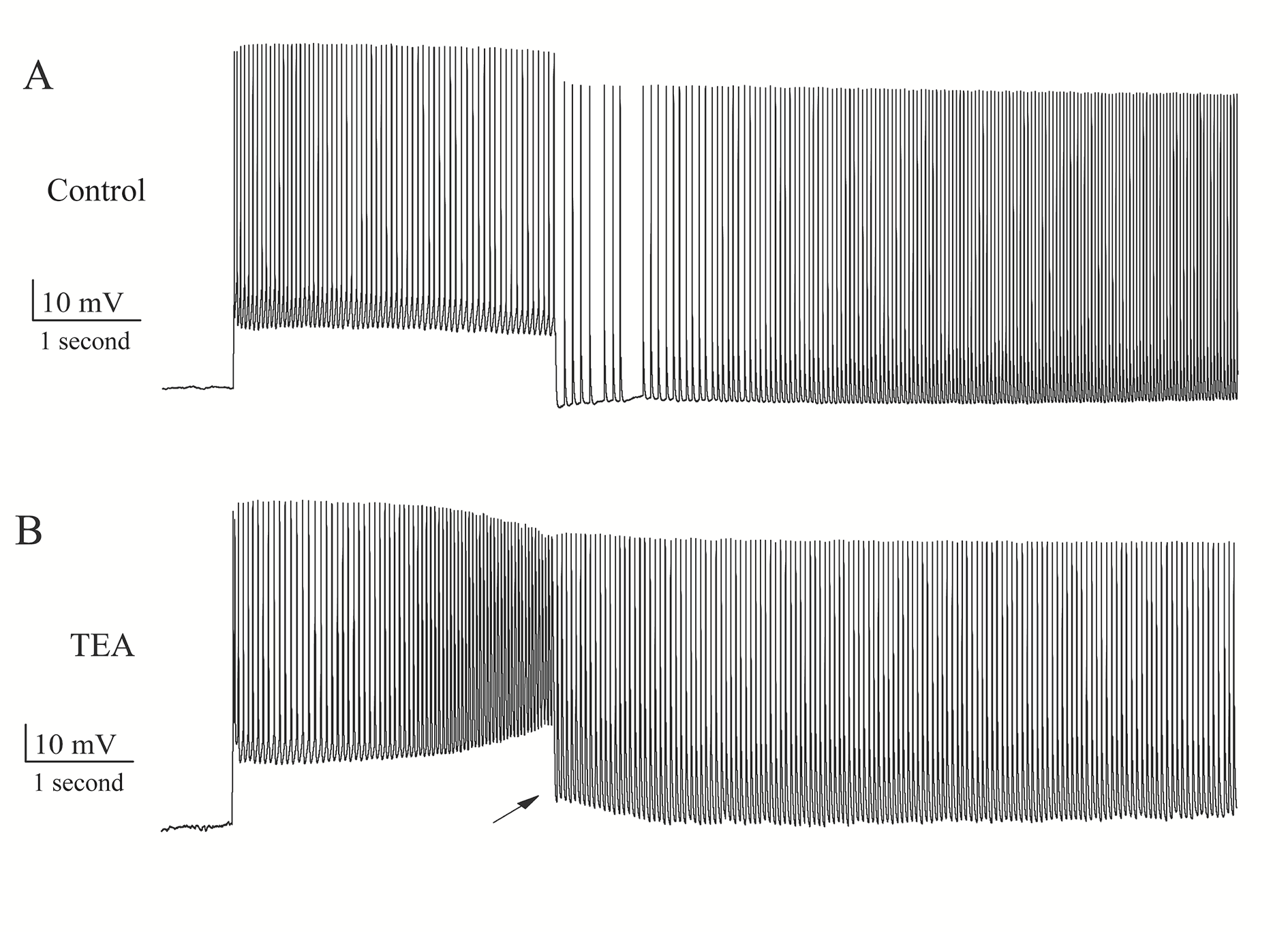


**Additional file three: Figure S3** AISS induction in GABAergic neurons does not require voltage-gated potassium channels. **A)** AISS is induced by their intensive activity under the control condition. **B)** After AISS disappears for six minutes, their intensive activity induces AISS in the presence of 40 mM TEA, a blocker of voltage-gated potassium channels. TEA effectiveness can be sure by observing the incomplete repolarization (indicated by arrow). Calibration bars are 10 mV and 1 second.
