# Supplementary material for: Voltage-independent sodium channels emerge for an expression of activity-induced spontaneous spikes in GABAergic neurons
Source: Mol Brain. 2014 May 20;7:38. doi: 10.1186/1756-6606-7-38 (PMC4039334; doi:10.1186/1756-6606-7-38)
Supplement: Additional file 4: Figure S4 — AISS induction in GABAergic neurons does not require voltage-gated low threshold calcium channels. A) AISS is induced by their intensive activity under the control. B) After AISS disappears for six minutes, their intensive activity induces AISS in presence of 100 μM mibefradil, a blocker of voltage-gated calcium channels that possess low threshold. Calibration bars are 10 mV and 1 second. [file 1756-6606-7-38-S4.doc]

**
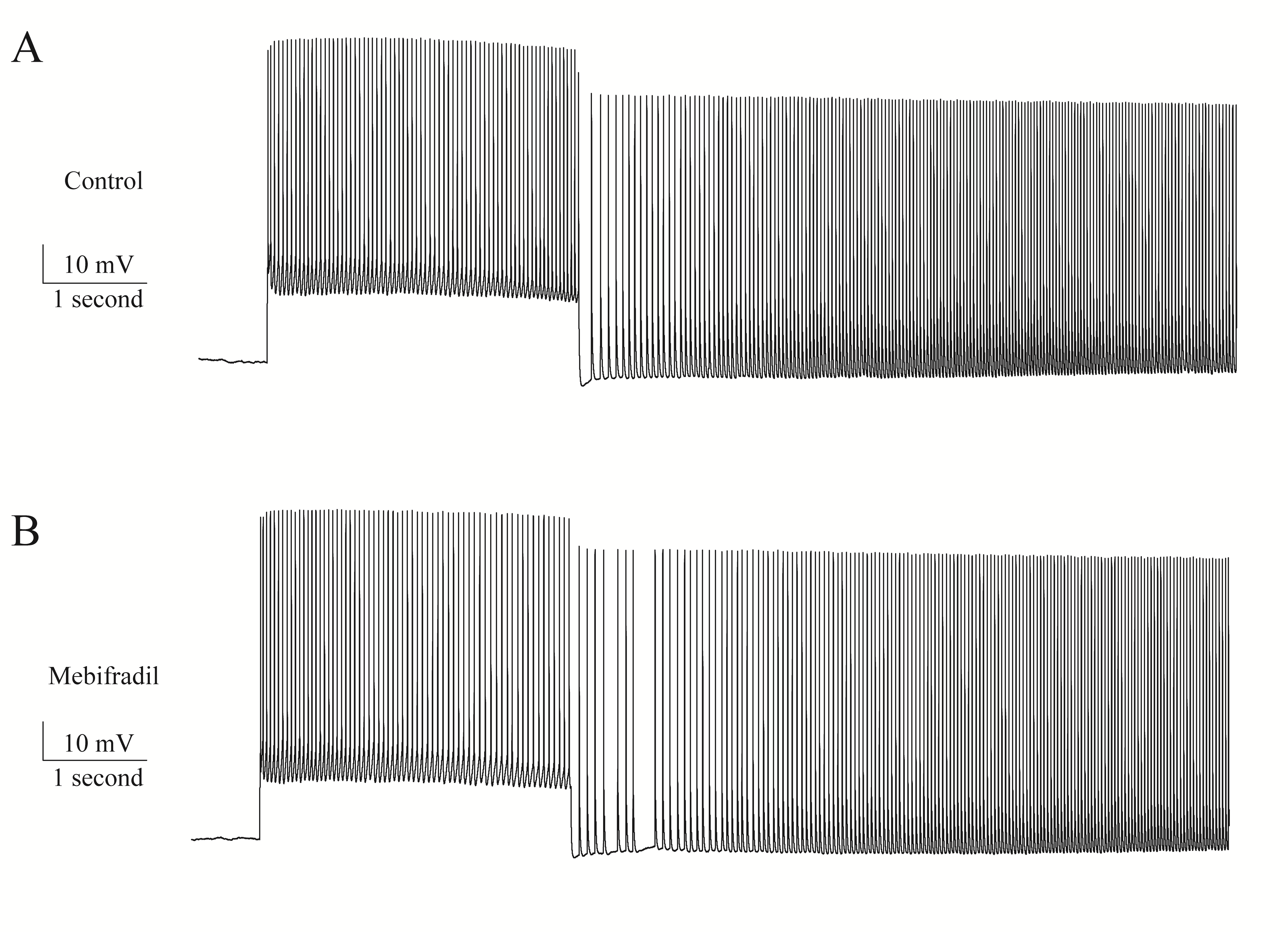
**

**Additional file four: Figure S4** AISS induction in GABAergic neurons does not require voltage-gated low threshold calcium channels. **A)** AISS is induced by their intensive activity under the control. **B)** After AISS disappears for six minutes, their intensive activity induces AISS in presence of 100 μM mibefradil, a blocker of voltage-gated calcium channels that possess low threshold. Calibration bars are 10 mV and 1 second.
