# Supplementary material for: Voltage-independent sodium channels emerge for an expression of activity-induced spontaneous spikes in GABAergic neurons
Source: Mol Brain. 2014 May 20;7:38. doi: 10.1186/1756-6606-7-38 (PMC4039334; doi:10.1186/1756-6606-7-38)
Supplement: Additional file 5: Figure S5 — AISS induction and expression are accompanied by the intracellular Ca2+ elevation in hippocampal GABAergic neurons, but blocked by preloading 1 mM BAPTA in these neurons. A) The level of intracellular Ca2+ was measured by loading fluo-3 into the recorded neurons through the recording pipette and by quantifying the fluorescent intensity under a laser scanning confocal microscope. Top panel shows the dynamic changes of fluorescent intensity in that the level of intracellular Ca2+ based on the binding of fluo-3 with Ca2+ is proportional to the spike trainings for evoking AISS. Bottom panel shows the process of AISS induction and expression. B) The intracellular preloading of BAPTA does not prevent AISS induction and expression. 1 mM BAPTA was included in the recording pipette. After the formation of whole-cell recording for 5 minutes, the depolarization pulses were injected into the neurons to induce AISS. The use of 1 mM BAPTA is based on the following considerations. 1) In general, the intracellular Ca2+ increases to 10-4~-5 during cell activation from 10-8 under the resting condition, and one BAPTA is able to bind two Ca2+ so that 1 mM BAPTA in the recording neurons should be able to buffer Ca2+ increase from intensive neuron activity. 2) This concentration of BAPTA was sufficient to block the functional plasticity induced in the neurons. 3) BAPTA is an acidic reagent. The high concentration of BAPTA in the recording pipettes may induce cellular acidosis. The high concentration of BAPTA will change the osmolarity of the recording neurons. This condition leads to the study not being physiological in nature. [file 1756-6606-7-38-S5.doc]

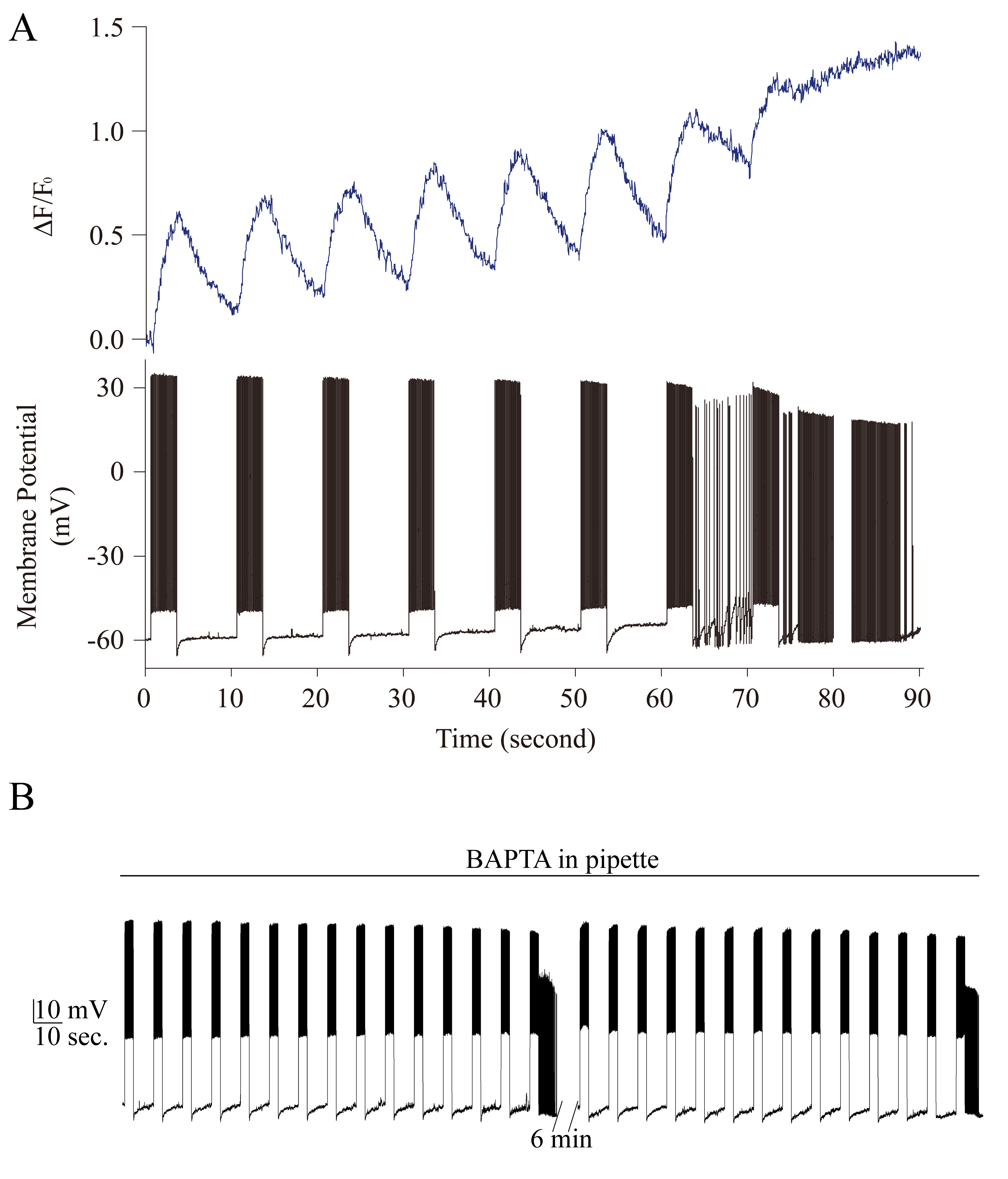


**Additional file five: Figure 5** AISS induction and expression are accompanied by the intracellular Ca^2+^ elevation in hippocampal GABAergic neurons, but blocked by preloading 1 mM BAPTA in these neurons. **A)** The level of intracellular Ca^2+^ was measured by loading fluo-3 into the recorded neurons through the recording pipette and by quantifying the fluorescent intensity under a laser scanning confocal microscope. Top panel shows the dynamic changes of fluorescent intensity in that the level of intracellular Ca^2+^ based on the binding of fluo-3 with Ca^2+^ is proportional to the spike trainings for evoking AISS. Bottom panel shows the process of AISS induction and expression. **B)** The intracellular preloading of BAPTA does not prevent AISS induction and expression. 1 mM BAPTA was included in the recording pipette. After the formation of whole-cell recording for 5 minutes, the depolarization pulses were injected into the neurons to induce AISS. The use of 1 mM BAPTA is based on the following considerations. 1) In general, the intracellular Ca^2+^ increases to 10^-4~-5^ during cell activation from 10^-8^ under the resting condition, and one BAPTA is able to bind two Ca^2+^ so that 1 mM BAPTA in the recording neurons should be able to buffer Ca^2+^ increase from intensive neuron activity. 2) This concentration of BAPTA was sufficient to block the functional plasticity induced in the neurons. 3) BAPTA is an acidic reagent. The high concentration of BAPTA in the recording pipettes may induce cellular acidosis. The high concentration of BAPTA will change the osmolarity of the recording neurons. This condition leads to the study not being physiological in nature.
